# Understanding Barriers to Disclosure of and Treatment for Substance Use During Pregnancy: A Narrative Synthesis of Literature

**DOI:** 10.1155/jp/6969976

**Published:** 2026-07-15

**Authors:** Nethmini Ranasingha, Helen Tosin Oni, M. Mofizul Islam

**Affiliations:** ^1^ School of Psychology and Public Health, La Trobe University, Bundoora, Victoria, Australia, latrobe.edu.au; ^2^ Department of Public Health, School of Psychology and Public Health, La Trobe University, Bundoora, Victoria, Australia, latrobe.edu.au

**Keywords:** barriers, disclosure, pregnant women, substance use, treatment

## Abstract

**Background:**

Pregnant women experience significant barriers to disclosing substance use and accessing treatment services, with serious implications for both maternal and fetal health. Despite the importance of this issue, no literature review has previously synthesised the available evidence. This review brings together current literature on the barriers to disclosure and treatment engagement among pregnant women who use substances.

**Methods:**

A systematic search was conducted across Ovid Medline, Embase, PsycINFO, Web of Science, Scopus, CENTRAL, PubMed, Google Scholar, ResearchGate, and Academia. Study quality was assessed using the Mixed Methods Appraisal Tool. Data extraction followed a predefined template, and data were analyzed using a narrative synthesis approach.

**Results:**

Nineteen studies were included in the analysis, which identified multiple barriers to prenatal substance use disclosure and treatment across personal, systemic, and financial domains. Barriers to disclosure were stigma, fear of judgment, and distrust of healthcare providers. Barriers to treatment encompassed structural challenges, including fragmented healthcare systems, limited provider knowledge and inconsistency in care, inadequate screening, and a shortage of specialised treatment facilities. Limited childcare facilities and financial constraints further reduced treatment engagement.

**Conclusions:**

The findings underscore the need for integrated care models that address these barriers and support early interventions to encourage disclosure and treatment uptake. Future research should examine how different groups of women are affected by these challenges to inform more targeted, culturally responsive, and equitable interventions.

## 1. Introduction

Prenatal substance use is a major public health concern with critical implications for both maternal and fetal health [1]. Globally, a study conducted by Popova et al. [2] estimated that about 10% of pregnant women consume alcohol during pregnancy. In Australia, approximately 28% of women report consuming alcohol during pregnancy [3]. Earlier data indicate that about one‐quarter of women continued to consume similar levels of alcohol both before and after becoming aware of their pregnancy [4]. Comparable prevalence has been reported internationally. For example, a recent U.S. study found that nearly one in five pregnant women used alcohol, tobacco, marijuana, or other substances [5, 6]. Countries adopt different policies, procedures, and protocols to manage the risks associated with prenatal substance use. In Australia, healthcare providers routinely screen pregnant women for alcohol and other drug (AOD) use and refer them to specialised AOD and perinatal support services where appropriate [7]. Healthcare providers are also mandated by legislation to identify and report risks to the unborn or newborn child to statutory child protection services (CPS).

Substances used during pregnancy can cross the placental barrier, potentially exerting teratogenic effects, the severity of which depends on the type of substance, dosage, frequency, and timing of exposure [8]. Prenatal substance exposure has been associated with a range of adverse perinatal outcomes, including fetal growth restriction, congenital anomalies, and neurodevelopmental impairments [9, 10]. Fetal alcohol spectrum disorders (FASD), for example, are characterised by craniofacial abnormalities [11], cognitive deficits, and behavioral impairments [12]. Prenatal tobacco exposure has been associated with intrauterine growth restriction, preterm birth, and reduced birth weight [13], and cannabis use during pregnancy has been linked to impaired neurodevelopment and an elevated risk of attention‐deficit/hyperactivity disorder [14].

The impact of prenatal substance use extends beyond fetal outcomes to significant maternal health risks. Pregnant women who use substances face an increased risk of severe maternal morbidity [15], including hypertensive disorders, gestational diabetes, and placental abruption [16]. Additionally, substance use during pregnancy is associated with a higher likelihood of miscarriage and stillbirth [17]. The burden on maternal healthcare systems is further exacerbated by prolonged hospitalizations due to substance‐related complications, such as opioid use disorder and withdrawal syndromes, highlighting the need for targeted interventions and comprehensive prenatal care [18].

Beyond the physiological consequences, prenatal substance use is frequently comorbid with psychiatric disorders. Pregnant women with substance use disorders are at heightened risk for co‐occurring mental health conditions, including major depressive disorder [19], generalised anxiety disorder [20], and posttraumatic stress disorder [21]. The bidirectional relationship between mental health disorders and substance use complicates treatment efforts, necessitating integrated care models that address both psychiatric and substance use conditions concurrently [1].

Despite its risks, pregnancy can serve as a powerful motivator for women to disclose substance use and engage with treatment services [22]. However, disclosure and treatment uptake remain limited, with many women facing barriers rooted in individual, social, legal, and healthcare contexts [23–25]. Sociocultural constructions of the “good mother” ideal frame pregnancy as a period of strict behavioral regulation and self‐sacrifice, where fetal needs are placed above maternal needs [26] and substance use is positioned as deviance from maternal norms and interpreted as evidence of being a “bad mother” [27]. This perceived deviance intensifies stigma, shame, and fear of judgment and can discourage women from attending antenatal care, disclosing substance use, or accessing treatment—particularly when disclosure is perceived to invite punitive responses or increased surveillance.

Existing research has tended to examine these barriers in isolation, and a comprehensive synthesis has been lacking. This review addresses this gap by integrating global evidence to identify the key barriers to disclosure and treatment engagement among pregnant women who use substances.

## 2. Method

### 2.1. Study Design

A narrative review methodology was employed, incorporating comprehensive database searches, study screening and selection, data extraction, quality appraisal, and narrative synthesis.

### 2.2. Inclusion and Exclusion Criteria

Studies were eligible for inclusion if they met the following criteria: (i) focused exclusively on pregnant women; (ii) examined substance use during pregnancy; (iii) investigated barriers to disclosure and/or treatment, including their impact on engagement with healthcare services; (iv) were published in English and aligned with the study objectives; and (v) employed qualitative, quantitative, or mixed‐method designs.

In addition, the review incorporated studies that examined barriers from the perspectives of both pregnant women and healthcare providers. Including provider perspectives was considered essential, as their clinical experiences offer valuable insights into the challenges faced by women who use substances during pregnancy.

Studies were excluded if they: (i) did not focus on substance use during pregnancy or on barriers to disclosure and treatment; (ii) failed to report outcomes relevant to the research question; (iii) presented overlapping data or duplicate publications; (iv) were case reports, dissertations, conference abstracts, or commentaries; or (v) were published in languages other than English, due to limited resources for translation.

### 2.3. Search Strategy

The search strategy was developed in collaboration with an information specialist from the lead author′s institution and refined with input from the coauthors. Searches were conducted between March 3 and April 11, 2024, across seven electronic databases: Ovid Medline (1946–present), Embase, PsycINFO, Web of Science, Scopus, CENTRAL, and PubMed, using a combination of relevant keywords and MeSH terms. These databases were selected for their comprehensive coverage of health and psychological sciences.

In addition, grey literature was identified through Google Scholar searches, and the reference lists of relevant articles were manually screened. Search strategies incorporated Boolean operators (“OR,” “AND,” “NOT”), phrase searching, truncation, wildcards, synonyms, abbreviations, and proximity operators. The process was supported by specialist librarians with expertise in systematic review search methodologies.

### 2.4. Study Selection

The study selection process involved two stages: title and abstract screening, followed by full‐text screening, conducted systematically using Covidence software. One author initially screened all titles and abstracts to assess eligibility, with Covidence providing structured evaluation, categorization, and documentation. To enhance rigour and minimise bias, a second author independently reviewed the screened records and provided an eligibility vote.

### 2.5. Data Extraction

A data extraction template was developed to ensure consistency in capturing relevant information from the final set of studies by an independent reviewer. To refine the categories, the template was pilot tested on a sample of studies. The lead author extracted data into the predefined template that included the title of the study, authors, years, study setting, aim, participants, sample size, study design, and outcomes. After the removal of duplicates, 2535 records were screened independently by two authors. Covidence flagged disagreements on 38 records as conflicts, which were resolved by a third author through detailed assessment and discussion, ensuring objective decision‐making. Forty full‐text articles were then retrieved and assessed against the predefined inclusion and exclusion criteria. Nineteen studies meeting all criteria were included in the final synthesis.

### 2.6. Quality Assessment

Quality assessment of the eligible studies was conducted using the Mixed Method Appraisal Tool (MMAT) 2018 version [28]. The MMAT ensures the validity and reliability of summarizing the quality of different methodologies, guaranteeing a consistent approach [28]. Two screening criteria are applied for all studies, followed by five criteria for qualitative, quantitative, and mixed methods. Each paper was assessed by two reviewers and discussed with the wider team to determine its suitability for inclusion and to resolve any conflicts.

### 2.7. Data Synthesis and Analysis

A narrative synthesis approach was adopted to integrate findings across the included studies. This method is particularly suited to literature reviews that encompass both qualitative and quantitative evidence, as it enables the reinterpretation and integration of diverse findings across multiple themes [29, 30].

Following established guidance for conducting narrative synthesis in systematic reviews [23], three iterative stages were undertaken: (1) developing a preliminary synthesis, (2) exploring relationships within and between studies, and (3) assessing the robustness of the synthesis.

The preliminary synthesis was independently conducted by the lead author, refined through iterative consultation with coauthors, and supported by structured data tabulation. Study characteristics and outcomes were tabulated to identify patterns and enable cross‐study comparison. Using Popay et al.′s [29] framework, participant narratives and author analyses from the included studies were inductively coded. Thematic coding was applied to categorise barriers systematically, followed by thematic analysis. An initial coding framework was developed inductively during the synthesis and refined as additional themes emerged. Vote counting was employed to capture the frequency of themes, indicating their relative prominence.

In the second stage, the lead author examined relationships within and across studies, considering variations in study design, outcomes, populations, and settings. When disagreements arose, they were resolved through review and adjudication by a third author.

Finally, the robustness of the synthesis was assessed through critical reflection and triangulation across the research team. The lead author′s background in psychology and public health, combined with the coauthors′ expertise in clinical practice, prenatal substance use research, and literature review, strengthened the interpretive process. The team engaged in regular discussions to critically examine assumptions, address potential biases, and reflect on their disciplinary perspectives, thereby enhancing the transparency and validity of the synthesis.

## 3. Results

### 3.1. Description of the Eligible Studies

**Figure 1:**
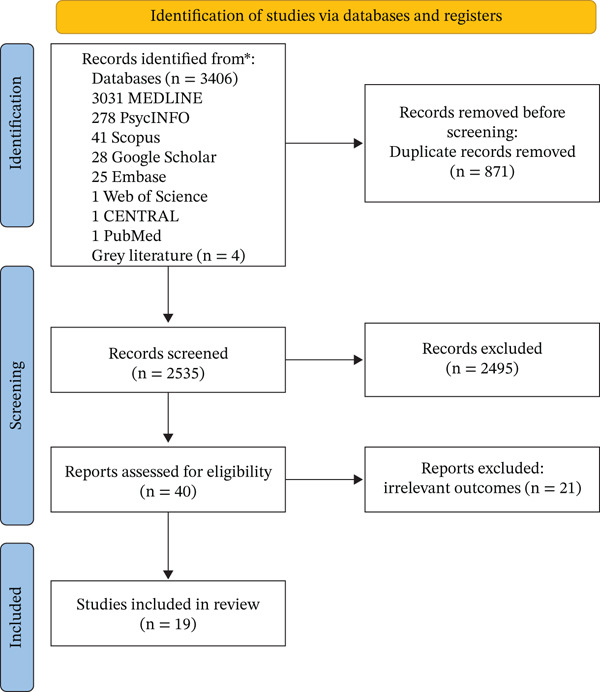
Flow chart presenting steps for selecting eligible studies.

A total of 3410 records were identified across seven databases, with an additional four records retrieved from grey literature sources. After removing 871 duplicates and excluding 2495 irrelevant studies, 40 full‐text articles were assessed for eligibility. Of these, 19 studies met the inclusion criteria and were included in the final review (Figure 1).

Among the included studies, the majority (*n* = 17) employed qualitative designs, using approaches such as grounded theory, iterative analysis, thematic analysis, situational analysis, and inductive content analysis. Two studies used quantitative designs, collecting data through questionnaires and reporting descriptive findings. Geographically, 15 studies were conducted in the United States, two in Australia, and one each in the United Kingdom and Slovenia (Table 1). Study settings ranged from prenatal clinics and community health centers to both public and private hospitals.

**Table 1:** Key findings of the studies reporting barriers to disclosure of and treatment for substance use during pregnancy.

Article name	Author(s) (year)	Country	Study aim	Participants	Study design	Identified barriers
Pregnant women and substance use: fear, stigma, and barriers to care	Stone [31]	United States	Gain a greater understanding of the way substance‐using women navigate the health and justice systems to avoid criminal justice consequences and to access needed health and social support resources.	30 women who were pregnant during the study or within the last 12 months	Qualitative	‐ Fear of detection
						‐ Fear of (CPS) involvement
						‐ Social isolation and denial of pregnancy
				Mean age: 28.5 years		
						‐ Limited health literacy, treatment accessibility and resources, and lack of transportation
				Ethnicity: White, African American, Hispanic, Native American, mixed/other		
				Setting: hospital		‐ Poor quality treatment
Factors that influence women′s disclosures of substance use during pregnancy: A qualitative study of ten midwives and ten pregnant women	Phillips et al. [32]	Australia	Examine the factors that motivate or act as barriers to disclosure of substance use by pregnant women.	20 (10 midwives, 10 pregnant women)	Qualitative	‐ Fear of CPS involvement
						‐ Inadequate rapport with healthcare
				Age: 18 years or older		‐ Dysfunctional family
						‐ Intimate partner violence and substance use
				Setting: hospital		
						‐ Inadequate knowledge and skills of providers
Psychometric properties of the prenatal opioid use perceived stigma scale and its use in prenatal care	Bann et al. [33]	United States	Examine the psychometric properties of the prenatal opioid use perceived stigma (POPS) scale and to assess the relationship of POPS scores to adequate prenatal care.	127 women who used opioids during pregnancy and whose infants participated in the outcomes of babies with opioid exposure	Prospective cohort	‐ Self‐stigma
				Age range: < 25–≥ 35 years		
				Ethnicity: Non‐Hispanic White non‐Hispanic Black Hispanic		
				Setting: hospital		
Social stigma and perinatal substance use services: Recognizing the power of the good mother ideal	Nichols et al. [34]	United States	Provider discourse on service provision to mothers with a substance‐exposed pregnancies and grounds the examination in ideological beliefs surrounding motherhood.	Providers who worked with pregnant mothers with substance use disorders	Qualitative	‐ Self‐stigma
						‐ Negative perceptions of healthcare providers
				Age range: 25–73 years		
				Ethnicity: Caucasian. Other participants identified as African American, Black, or human		
				Setting: community		
Complex calculations: How drug use during pregnancy becomes a barrier to prenatal care	Roberts and Pies [35]	United States	Explore women′s perspectives on barriers to prenatal care and understand the processes through which drug use and its associated actors during pregnancy become barriers.	38 women of racially/ethnically diverse backgrounds and low‐income group who were using alcohol and drugs	Qualitative	‐ Inadequate rapport between healthcare providers
						‐ Social isolation
						‐ Fear of CPS involvement
				Setting: community‐based treatment service		‐ Limited transportation and financial constraints
						‐ Inadequate treatment facilities
In their own words: A qualitative study of factors promoting resilience and recovery among postpartum women with opioid use disorders	Goodman et al. [36]	United Kingdom	Explore the experiences of postpartum women with OUD who engaged in both substance use treatment and maternity care during pregnancy, to learn about barriers and facilitators that contributed to the ability to achieve recovery in the face of personal and structural challenges.	10 postpartum women in sustained recovery	Qualitative	‐ Fear of CPS involvement
						‐ Inadequate rapport between providers
				Mean age: 28 years		
				Ethnicity: non‐ Hispanic White or multiracial (White/Native American)		
						‐ Financial constraints
						‐ Limited transportation
				Setting: community‐based treatment service		
The experience of pregnancy, childbirth and motherhood of drug‐using women	Mejak and Kastelic [37]	Slovenia	Explore and better understand the experiences of women using illicit drugs during pregnancy, childbirth and motherhood, and recognise their needs and the obstacles they have to face.	15 pregnant women who used opioids.	Qualitative	‐ Limited social support
						‐ Stigma
				Age range: 24–34		‐ Fear of CPS involvement
						‐ Late discovery of pregnancy
				Setting: clinic		‐ Lack of integrated care
						‐ Inadequate knowledge of prenatal substance use
Barriers to women′s disclosure of and treatment for substance use during pregnancy: A qualitative study	Oni et al. [38]	Australia	Explore barriers women encountered in disclosing substance use and accessing substance use treatment in pregnancy.	15 participants who reported a history of substance use during one or more of their pregnancies.	Qualitative	‐ Fear of CPS involvement
						‐ Self‐stigma, social stigma
						‐ Negative perceptions from providers
						‐ Inadequate support from providers
				Age range: 26–53 years		
						‐ Limited treatment options
				Setting: community		‐ Difficulty in everyday life and meeting daily needs
The labor and birth experience of women with opioid use disorder: A qualitative study	O′Rourke‐Suchoff et al. [39]	United States	To describe the experience of pregnancy and childbirth from the perspective of women with opioid use disorder	Nine women with opioid use disorder	Qualitative	‐ Fear of CPS involvement
						‐ Inadequate support from providers
				Age range: 22–35 years		
				Setting: hospital		
Health care providers′ perceived barriers to screening for substance use during pregnancy	Pentecost et al. [40]	United States	Explore health care providers′ perceived barriers to conducting standardized screening processes for substance use during pregnancy.	Healthcare providers of 12 women from two hospitals, including obstetricians/gynecologists, nurse practitioners, physician assistants and nurse‐midwives.	Survey	‐ Inadequate prenatal substance use screening
						‐ Fear of CPS involvement
						‐ Lack of integrated care (lack of referral resources, lack of organizational structure)
Secrecy versus disclosure: Women with substance use disorders share experiences in help seeking during pregnancy	Paris et al. [41]	United States	Examine the process of how and why pregnant women with SUDs choose to disclose or not disclose their substance misuse to their providers when seeking prenatal care	21 participants residing in a Northeast U.S. urban area	Qualitative	‐ Fear of CPS involvement
						‐ Self‐stigma
						‐ Fear of being judged by providers
				Age range: 21–44 years		
				Ethnicity: White, Black, Latina		
Conceptualizing stigma in contexts of pregnancy and opioid misuse: A qualitative study with women and healthcare providers in Ohio	Syvertsen et al. [42]	United States	Examine how stigma shaped the experiences of pregnant women with histories of opioid misuse and the perspectives of healthcare providers who work with these women.	28 women with histories of opioid misuse who were pregnant or recently gave birth and 18 healthcare providers in Ohio.	Qualitative	‐ Enacted stigma
						‐ Structural stigma
						‐ Anticipated and internalised stigma
				Mean age: 30 years		
				Ethnicity: White, non‐White		
				Setting: community		
“The elephant in the room;” a qualitative study of perinatal fears in opioid use disorder treatment in Southern Appalachia	Leiner et al. [43]	United States	Examine facilitate greater understanding of perinatal OUD patients′ fears, concerns, needs, and priorities and provide lessons for reproductive health and substance use treatment providers.	27 pregnant and postpartum patients	Qualitative	‐ Fears of social services involvement
				Mean age: 28 years		‐ Anxious about child removal
				Ethnicity: 26 White and 1 Black		
				Setting: community‐based treatment program in Southern Appalachia		
Treatment for substance use disorders in pregnant women: Motivators and barriers	Frazer et al. [44]	United States	Identify the motivators and barriers to substance use disorders treatment during pregnancy.	20 women in treatment at The Center for Addiction and Pregnancy (CAP) at comprehensive care facility for pregnant and parenting women	Qualitative	‐ Homelessness
						‐ Lack of information or misconception about the availability of the treatment
						‐ Fear of legal action affecting custody of children
				Age range: 22–38 years		‐ Not wanting to leave the children or partner at home
				Ethnicity: non‐Hispanic White, non‐Hispanic Black		
						‐ Stigma and judgment associated with SUD treatment
						‐ Limited availability of appropriate treatment facilities
						‐ Lack of childcare, affordable and convenient transportation
Personas of pregnant and parenting women with substance use and their barriers and pathways to system engagement	Foti et al. [45]	United States	Identify pathways of and barriers to perinatal system and service engagement in Florida.	118 healthcare professionals who routinely address perinatal substance use.	Qualitative	‐ Shame and guilt
						‐ Fear of CPS involvement
						‐ Poor treatment quality
						‐ Limited treatment options and facilities
						‐ Housing instability and financial struggles
						‐ Limited transportation
						‐ Dysfunctional family and lack of support
Pregnant people′s experiences discussing their cannabis use with prenatal care providers in a state with legalised cannabis	Woodruff et al. [46]	United States	Explore the experiences of pregnant women who used cannabis during pregnancy in California after the legalization of recreational cannabis, specifically focusing on their interactions with healthcare providers regarding cannabis use.	33 pregnant or postpartum women who used cannabis during pregnancy	Qualitative	‐ Fear of CPS involvement
						‐ Fear of being judged by healthcare providers
						‐ Providers′ lack of initiation of discussions about cannabis use
				Age range: 18–45 years		
						‐ Loss of trust among pregnant women and the healthcare provider
				Ethnicity: Black, Latina, White, American Indian, more than one race, Asian/pacific Islander		
				Setting: community‐ and clinic‐based recruitment		
Pregnancy‐ and parenting‐related barriers to receiving: A multi‐paneled qualitative study of women in treatment, women who terminated treatment, and the professionals who serve them	Apsley et al. [47]	United States	Assess the parenting and pregnancy‐related barriers women with opioid use disorder face in seeking medication for opioid use disorder (MOUD) treatment.	‐12 SUD treatment professionals and 10 criminal justice professionals who work with women who use MOUD.	Qualitative	‐ Lack of childcare
						‐ Fear of losing custody of children
						‐ Temporary separation concerns
						‐ Perceived history by physicians
						‐ Limited resources in rural areas
				20 women with lived experience of MOUD.		‐ Navigating multiple facets of the healthcare system
				Mean age: 37 years §		
				Ethnicity: African‐ American, Caucasian, Biracial		
Barriers to care for pregnant women seeking substance use disorder treatment	Qato [48]	United States	Explore the role that stigma, racism and other sociodemographic, structural, and political determinants play in challenging access to healthcare for vulnerable pregnant women who use substances.	Five women and two providers	Qualitative	‐ Lack of access
						‐ Fear of engagement with child social services
						‐ Co‐occurring mental health illness
						‐ Lack of housing
						‐ Fear of stigma and of being judged by providers
						‐ Lack of awareness about available options
Extrinsic barriers to substance abuse treatment among pregnant drug dependent women	Jessup et al. [49]	United States	Examine extrinsic barriers to substance abuse treatment among pregnant and parenting women enrolled in residential perinatal substance abuse treatment programs in Northern California.	15 pregnant women	Qualitative	‐ Fear of losing custody of children
				Mean age: 30.2 years		‐ Disrupted and delayed residential treatment entry
				Ethnicity: Black, White, Latina, Native American		
						‐ Limited childcare facilities
						‐ Preadmission requirements for detox
				Setting: community treatment program		‐ Domestic violence and partners′ substance abuse
						‐ Limited Medicaid coverage

Abbreviations: CPS, Child Protection Services; MOUD, medications for opioid use disorder; OUD, opioid use disorder; SUD, substance use disorder § for 20 women with lived experience of MOUD.

As per the MMAT criteria, 15 studies demonstrated high quality (Tables in Supporting Information (Available here)). However, four studies had issues in two critical areas: (i) incoherence among data sources, collection methods, analysis, and interpretation; and (ii) moderate coherence between their findings and the broader research context.

### 3.2. Characteristics of the Participants in the Included Studies

The participants in the included studies were pregnant women with a history of substance use, women actively using substances at the time of data collection, and healthcare providers. Twelve studies explored barriers to disclosure and treatment during pregnancy from the perspectives of pregnant women [31–33, 35–39, 41–44, 46]. Two studies focused exclusively on the perspectives of healthcare providers [34, 40], whereas five studies examined both perspectives [32, 45, 47, 48].

Across the 19 studies, participants were predominantly in their late twenties to early 30s, often mothers of one to three children. The most reported substances were opioids, followed by tobacco, alcohol, and cocaine. Participants were frequently from low socioeconomic backgrounds, characterised by financial hardship, unstable employment, and in many cases, homelessness.

### 3.3. Barriers to Disclosure of Substance Use During Pregnancy

Two overarching themes were identified as barriers to the disclosure of substance use during pregnancy: (i) stigma and (ii) fear and distrust.

#### 3.3.1. Stigma

Stigma was the most prominent barrier to disclosure, reported in 11 studies [33–35, 37, 38, 41, 42, 44–46, 48]. Feelings of guilt and shame, reinforced by societal views of substance use in pregnancy as a moral failing, led many women to hide their use [34, 35, 39, 41, 45]. Women expressed an overwhelming sense of responsibility for potential harm to their unborn child, reinforcing secrecy and reluctance to disclose substance use to healthcare providers. Here are two quotes from women:

“That guilt of knowing that you′ve used causes you to know 9 times out of 10 [it] will affect your baby in some way, form or another.” [35].

“I was secretive, … I did use a few times, to tell you the truth, but I was like really secretive of it… I didn′t tell anyone because of the stigma and the guilt. I never appeared off my head or anything, so I never got tested so that they just thought I was off it.” [38].

In contrast, some women reported that guilt acted as a motivator to seek prenatal care, aiming to mitigate harm to their child.

“[I′m glad that I went to the doctor during my pregnancy] because I was able to see that my child was growing healthy and that I was doing all the right things.” [38].

Beyond guilt, social perceptions that depict substance use during pregnancy as an irreversible moral failing intensified feelings of shame, as indicated in six studies [31, 34, 38, 39, 41, 48]. Participants frequently encountered harsh judgment from both healthcare providers and society for failing to conform to the idealised notions of “good mothering” [34]. These judgments undermined their capacity for responsible parenting and reinforced a feeling of inadequacy, with many likening their stigma to carrying a symbolic “scarlet letter” [41]. The following quote illustrates this observation:

“Some doctors stuck their nose up at me…pretty much calling me a bad parent because I was using drugs… I stopped seeing them.” [38].

Some healthcare providers made judgments about women′s parenting abilities based on their substance use history, reinforcing shame and withdrawal from care, as the following quotes suggest:

“The doctors were like, I′ve never seen a mother like you. I′ve never seen a mother like you, especially a recovering addict blah blah blah.” And I′m like, “hold up! So, you′re saying a recovering addict can′t advocate for her son? Can′t stick up for her son? And can′t be adamant about what goes on?” [39].

“I feel like I was being judged. I just had that feeling because that′s the energy I felt like I received from one of the … They don′t know my story. They don′t know what′s going on with me. I just feel like I was just being judged, so it made me really feel uncomfortable and it hurt my feelings because I felt like I wasn′t being handled with respect.” [39].

Healthcare providers have acknowledged the presence of stigma in clinical environments [32, 34, 48, 50]. Some providers expressed hesitancy in their interaction with pregnant women using substances, fearing they might unintentionally reinforce stigma. For instance, a provider states:

“Honestly, I was fearful when I started seeing that population, not because I was afraid of …. um they′re very judged and I didn′t want them to feel like I was judging them or prying them. So, in the beginning, I kind of kept my distance.” [34].

Despite these challenges, some providers recognised the need to adopt a nonjudgmental, supportive approach to care. They endeavored to foster a safe environment for disclosure and healthcare engagement [32, 34, 42]. The following quotes are illustrated in two studies:

“Letting them know that parenting is hard but that they can do it… Look at how much they′ve done already for their baby to get into treatment.” [34].

“I think that it is all up to me in terms of developing that rapport and making her feel comfortable and making her feel safe as to how much information I get” [32].

#### 3.3.2. Fear and Distrust

In 14 studies, the fear of CPS′ involvement was identified as a barrier to the disclosure of substance use among pregnant women [31, 32, 35, 37–39, 41–44, 46–49]. Four studies highlighted that CPS involvement was perceived as a threat due to its potential long‐term consequences, including loss of child custody, family disruption, and psychological distress [35, 37, 43, 48]. The fear was often rooted in previous interactions or observations of CPS interventions, leading to avoidance of healthcare services [43]. Below are illustrative quotes from pregnant women in one of the studies:

“I was scared coming here because I thought it′s gonna be immediate Social Services [involvement]. I [thought I] would never see my child again.” [43].

“Because I was smoking marijuana, they told me CPS would get involved if they found THC in my system. So, I didn′t go to any care for her, none.” [43].

Beyond healthcare contexts, studies found that the psychological burden associated with CPS involvement had a profound effect on the social lives of pregnant women with substance use [31, 43]. Some women withdrew from social interactions entirely to minimise perceived risks.

“I had stopped talking to everyone, period. because I didn′t want the wrong person to go over there and say something. I didn′t want them to go do that and I didn′t know who to trust, so I wasn′t saying anything…” [31].

A key factor exacerbating this fear was the role of third‐party reporting, which further eroded trust in healthcare and social support systems. Some of the women alluded that reports to CPS were sometimes motivated by personal retaliation rather than genuine concern for the child′s welfare; as Stone [31] reported, a mother was involved in a dispute with another woman and falsely reported her to CPS. However, in another instance, an abusive expartner of a woman sought retribution after their separation by disclosing her substance use while pregnant:

“I broke up with my abusive boyfriend, and in retaliation, he called CPS and told them I was pregnant and smoking marijuana.” [31].

Additionally, a lack of understanding of CPS policies and mandatory reporting requirements intensified anxiety among pregnant women. Uncertainty about which substances or behaviors would trigger CPS involvement led many to refrain from seeking clarification, fearing that inquiries might raise suspicion. For instance, when a woman was asked if she feared CPS involvement, she said:

“I guess I would say no, only because nothing like that had happened before. So I hadn′t seen it and I never really knew anybody who it had happened to, either, so it really almost didn′t even seem like a possibility, I mean.” [31].

However, contrary to the prevailing fear, several studies [35, 44, 46] found that some pregnant women perceived CPS involvement as a catalyst for behavioral change. The prospect of losing custody served as a motivator to engage in prenatal care, either as a demonstration of commitment to their child′s well‐being or as a means of regaining custody:

“[Fear of CPS] made me not want to go, but because I was high risk, there was a greater chance of, I care more about my son being ok…so, I never missed a [n] appointment” [35].

“One of the reasons I went to was because I didn′t want them to take the baby away.” [35].

### 3.4. Barriers to Treatment for Substance Use During Pregnancy

**Figure 2:**
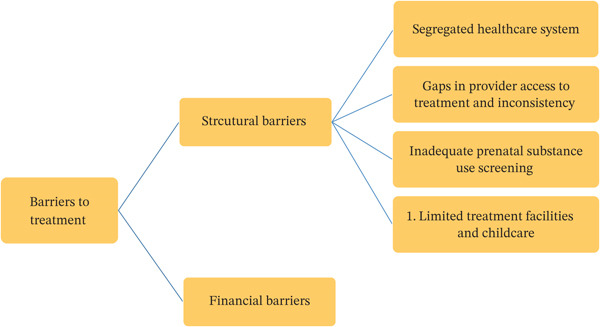
Barriers to treatment of substance use during pregnancy.

The barriers to seeking treatment for substance use during pregnancy were divided into two main categories: structural and financial. Structural barriers included segregated healthcare systems, gaps in provider access and treatment consistency, inadequate prenatal substance use screening, and limited treatment facilities and childcare options (Figure 2).

#### 3.4.1. Structural Barriers

##### 3.4.1.1. Segregated Healthcare System

Three studies highlighted the fragmented nature of the healthcare system as a significant barrier to treatment for pregnant women [42, 47, 48]. Syvertsen et al. [42] highlighted that some women were dismissed from their treatment programs due to a lack of coordination among healthcare providers. The disconnection between addiction specialists and obstetric providers created a gap in care, as each group expressed discomfort in addressing the other′s area of expertise. A healthcare provider described this divide with the following statement:

“OBs, in general, are frightened of women who have addiction issues, and addiction specialists are frightened of pregnant women” [42].

This lack of integration resulted in significant challenges for pregnant women, who were forced to navigate multiple, uncoordinated systems for prenatal, postnatal, and addiction treatment. The complexity of managing these disparate services often led to disengagement from care [47]. Here is another quote from a healthcare provider that highlights the overwhelming nature of these challenges:

“Oh, I need housing, and I don′t even know where to start. Or ‘Who do I talk to? Where do I go?’ If they′re just dealing with that and trying to navigate the system on their own, I think it′s very easy for them just to give up” [48].

##### 3.4.1.2. Gaps in Provider Access to Treatment and Inconsistent Care

Four studies highlighted gaps in provider access and inconsistent care as major structural barriers [39, 42, 44, 45]. A lack of healthcare professionals who are willing and able to provide addiction treatment, particularly to pregnant women, resulted in long wait times, financial barriers, and delayed care [42]. Women seeking buprenorphine treatment reported immense difficulty finding available and affordable providers [42]. Below is an illustrative quote from a pregnant woman:

“I had called so many doctors. Like a list of 10 or 15 doctors, and they either didn′t take my insurance or wanted hundreds of dollars even to get in to see them…. Or they did not have openings for months.” [42].

The barrier was exacerbated further by the hesitation of some providers to provide medication to pregnant women. A study focused on neonatal abstinence syndrome (NAS) in Ohio suggested that concerns over possible risks or liability prompted several practitioners to refer patients to other healthcare providers instead of providing treatment. Below is an illustrative quote from a healthcare provider in one of the studies:

“I know our doctor here, he won′t treat—he doesn′t see pregnant patients. If I have a female in my caseload on Suboxone maintenance and she becomes pregnant, I′ve got to transfer her out.” [42].

Furthermore, even where women had access to providers, the guidance and information women received were inconsistent, resulting in confusion and distrust. Many women obtained conflicting advice from healthcare professionals regarding the most effective course of treatment during pregnancy [37, 39, 45]. This, in turn, steers some pregnant women away from treatment. Below is an illustrative quote from a pregnant woman:

“My gynaecologist and social worker advised detoxification, while the other medical staff said there was no need to stop using methadone. I was very confused, because even the staff had no idea what was better. I trusted the drug specialist and was stable on methadone during the entire pregnancy, but I worried that the social worker would not accept my decision.” [37].

##### 3.4.1.3. Inadequate Prenatal Screening for Substance Use

Inadequate screening during the prenatal period was another barrier to effective care [32, 34, 37, 40]. Healthcare providers may not have the training or comfort to ask pregnant women about their substance use. Pentecost et al. [40] found that almost 33% of providers did not have adequate training and comfort in discussing substance use with pregnant women. Midwives often performed inconsistent screenings, relying on subjective factors like age or religion rather than standardised assessments [32]. Similarly, Woodruff et al. [46] noted that a lack of proper screening and knowledge led to negative assumptions about substance use by pregnant women. Here is a quote from a pregnant woman who expressed frustration about inadequate screening:

“He would probably be the dumbest doctor I′ve ever met if he is not aware that I smoke weed because it′s going to show up in my urine and my blood work.” [46].

##### 3.4.1.4. Limited Treatment Facilities and Childcare

Inadequate treatment facilities and limited resources were significant barriers to care [31, 38, 45]. A recurring challenge was access to withdrawal treatment despite women demonstrating urgent needs. One pregnant woman described her frustration with being refused care:

“And even when I went to [the local hospital] and said, ‘…Can you guys watch me while I detox?’ and they said no… So, you′re going to send me home to have a miscarriage, then…” [31].

Inadequate detoxification services also led some women to either continue using substances or reluctantly accept treatments they were uncomfortable with, such as methadone maintenance treatment (MMT). The following quote demonstrates a woman′s frustration with being forced into MMT due to the absence of alternative options:

“I didn′t want to go on MMT… I was begging not to go on that [MMT], but there′s no other option.” [38].

Moreover, many women faced challenges in finding treatment facilities that would accept them, with some facilities requiring patients to have already completed detoxification or be enrolled in MMT. One woman shared her experience of being turned away:

“None of them would accept pregnant women unless I were already detoxed or on methadone maintenance.” [31].

Limited access to childcare facilities while undergoing treatment further complicated access to care. Pregnant women often require intensive treatments that demand significant time commitments, and the lack of childcare options makes it difficult for them to engage fully in treatment [44, 47]. Here is a quote from a woman:

“I wish that there were more programs for my kids to do stuff closer to me because I don′t have childcare.” [47].

Healthcare providers also recognised the insufficiency of available treatment resources. Although pregnant women are a priority group for substance use treatment, long waitlists for care persist [45]. Here is a quote about the long waitlist:

“Technically, pregnant folks are the priority for both mental health and substance abuse, but the waitlists are long” [45].

##### 3.4.1.5. Financial Barrier

Financial barrier was a major obstacle to accessing treatment [35, 36, 42, 47]. Financial constraints, especially for vulnerable populations, limit the ability to participate in treatment. The following quote illustrates how homelessness prevented a woman from accessing prenatal care:

“I had always intended on going and having prenatal care, but the first blocker was the welfare department and getting Medi‐Cal… They had denied me Medi‐Cal because I was homeless [and therefore didn′t have an address]” [35].

For women enrolled in opioid replacement therapy, the long‐term cost of treatment raised concerns [45]. For instance, here is how a woman expressed her worry about the potential loss of Medicaid coverage and the financial strain it could impose:

“With the methadone, I do have my Medicaid that pays for it, and I do sometimes worry like, ‘What if that gets cut off?’ Because it′s expensive. But I would just have to find – I would have to find a way to pay for it” [45].

Additionally, the financial burden of childcare posed challenges for pregnant women needing intensive treatment, such as inpatient or intensive outpatient care [47]. Healthcare providers noted that women often could not afford the time or resources for necessary childcare during these treatments:

“Often, at the depths of treatments that they need is sometimes in‐patient treatment or intensive outpatient treatment that requires a lot of time. That time isn′t afforded to them because they also can′t afford to have childcare during that time” [47].

## 4. Discussion

This review identified several barriers to the disclosure and treatment of substance use among pregnant women. Disclosure was primarily hindered by stigma, fear, and distrust, whereas treatment was limited by structural and financial barriers. Structural barriers included fragmented healthcare systems, restricted provider access, inconsistencies in care, inadequate prenatal screening, and a shortage of treatment facilities and childcare support. Importantly, barriers to disclosure and treatment were closely interrelated. For instance, reluctance to disclose substance use reduced opportunities for referral to care, whereas limited access to treatment further discouraged women from revealing their substance use.

Stigma is a major barrier to disclosure of substance use during pregnancy, reinforced by negative stereotypes and judgmental attitudes in both social and healthcare settings [33, 34]. Such stigma fosters shame, self‐blame, and psychological distress, often leading to disengagement from care [51, 52]. Societal expectations of maternal responsibility further intensify moral judgments, whereas labels such as “recovering addict” can undermine women′s confidence in their maternal roles [34, 38, 41, 53].

Stigma within healthcare settings also plays a substantial role, as some providers expressed reluctance to engage with pregnant women who use substances [32, 34, 48, 50]. This stigma influences patient–provider interactions, shaping whether women feel supported or judged [54]. However, findings also suggested that a feeling of guilt, which is linked to internalised stigma, may influence some women to disclose substance use and seek prenatal care as a means of minimizing harm to their infant [35, 38]. Despite concerns about potential discrimination, the desire for a healthy pregnancy may encourage engagement with healthcare services, aligning with previous research highlighting the complex interplay between stigma, disclosure, and healthcare access [55].

Although CPS is intended to protect children from abuse or neglect, its involvement often deters pregnant women from disclosing substance use. Fears of custody loss, family disruption, and psychological distress were frequently reported as barriers to seeking support [31, 32, 35, 37–39, 41–44, 46–48]. Women′s fear and distrust stem from the legal and custodial consequences and the inconsistencies in CPS interventions, which vary across jurisdictions and are often influenced by provider discretion and subjective assessments [31, 43]. These types of punitive policies surrounding prenatal substance use risk erode trust in healthcare providers and discourage engagement with maternity care and treatment [56]. Despite these concerns, findings suggested that for some women, the threat of CPS involvement serves as a catalyst for behavioral change, motivating reductions in substance use or efforts to strengthen parenting skills [35, 46]. These findings highlight the complex and context‐dependent role of CPS, underscoring the need for sensitive and balanced approaches in case management.

From a treatment perspective, the healthcare system remains mostly fragmented, forcing pregnant women with substance use disorders to navigate disconnected services without adequate support [42, 47]. Evidence shows that integrated care reduces complications such as preterm birth and prolonged infant hospitalization and improves treatment engagement [36]. Yet, poor coordination between addiction specialists and obstetric providers, driven by limited training and provider hesitancy, continues to delay care and heighten risks for both mothers and infants [57].

Limited treatment options further restrict access to patient‐centered care. The lack of detoxification services and alternative evidence‐based treatments often forces women into treatment pathways misaligned with their preferences, undermining autonomy and leaving many feeling coerced [31, 38]. Treatment accessibility is further constrained by a shortage of clinicians trained to provide medication‐assisted treatment (MAT), such as methadone and buprenorphine. Complex referral systems, financial barriers, and provider reluctance—often fueled by misconceptions about the risks of pharmacotherapy during pregnancy—further limit uptake. This occurs despite strong evidence of MAT′s safety and effectiveness in reducing opioid use and improving maternal and neonatal outcomes [42, 44, 58, 59]. These barriers reflect broader systemic issues in policy, service integration, and resource allocation to treatment engagement.

The review also highlights the impact of healthcare provider bias on access to treatment and care for pregnant women who use substances. A lack of standardised and nonjudgmental assessments points to both knowledge gaps and deeper systemic issues in maternal healthcare. Studies show that, in the absence of clear guidelines and adequate training, providers often rely on subjective factors, such as age or religious background, instead of evidence‐based criteria when assessing substance use during pregnancy [34, 37, 40]. Cultural biases within healthcare settings can lead to women being dismissed or scrutinised based on personal judgments rather than clinical needs [60, 61]. Inconsistent screening delays early detection and intervention while further eroding trust in healthcare systems [32, 46].

Addressing these challenges requires coordinated and multidisciplinary care models that integrate maternity and addiction services, expand access to evidence‐based treatments and build relational safety through consistent, culturally safe engagement. Midwifery continuity of care (CoC) is one such model aligned with these aims, enabling women to receive care throughout pregnancy, birth, and the postnatal period from a known midwife or small team [62]. This approach has been associated with improved maternal satisfaction, greater engagement, and stronger trust in healthcare providers by upholding women′s autonomy and fostering a sense of safety.

## 5. Limitations

This review synthesised existing literature on barriers to disclosure and treatment for pregnant women with substance use; however, several limitations should be acknowledged. First, only English‐language studies were included, potentially excluding relevant evidence published in other languages. Second, the majority of included studies were conducted in the United States, which may limit generalizability to countries with different healthcare systems, policies, and cultural contexts. The U.S. health system, characterised by fragmentation, insurance‐based access, and workforce shortages [63], likely amplifies barriers such as service gaps and provider inaccessibility. Although many of the identified barriers are relevant across settings, some may be uniquely tied to the U.S. healthcare and legal environment. Future research should examine these issues across diverse international contexts to strengthen the global evidence base.

The review′s reliance on a limited number of databases may have resulted in the omission of relevant studies. In addition, the predominance of qualitative research, while offering rich insights, constrains the ability to estimate the prevalence of barriers across broader populations. The robustness of findings was also dependent on the quality of synthesis in the included studies. The review protocol was not formally registered; however, the predefined eligibility criteria and independent screening by expert reviewers ensured rigour.

The absence of demographic‐specific analyses limits understanding of how barriers to disclosure and accessing treatments may differ across subgroups such as age, ethnicity, level of education, socioeconomic status, geography, and parity, highlighting the need for further research in these areas. For example, ethnicity may influence experiences of surveillance, trust in healthcare systems, and fears related to involvement of CPS. In particular, racialized communities may experience heightened monitoring and intervention within these systems, which can create additional barriers to disclosure and care.

Finally, the included studies have conducted in various settings like hospitals, substance treatment services, and community programs, with pregnant women who predominantly engaged with services and systems. Therefore, the perspectives and experiences of pregnant women who do not access or engage with any type of substance use services can be varied, potentially due to heightened stigma and structural and financial barriers, limiting the ability to capture the experience of those encountering the significant barriers to disclosure and care.

## 6. Conclusion

This review highlights multiple barriers to disclosure and treatment of substance use during pregnancy, including stigma, fear, and distrust, particularly of CPS, as well as structural and financial obstacles. These barriers are complex and interrelated, underscoring the need for comprehensive, collaborative approaches that engage women, healthcare providers, and support services. Future research should explore demographic‐specific experiences to better understand how these challenges are perceived and experienced across diverse groups.

## Author Contributions

Nethmini Ranasingha: data curation, formal analysis, investigation, methodology, project administration, visualization, and writing—original draft, writing. Dr. Helen Tosin Oni: methodology, supervision, and writing—review and editing. Dr. M. Mofizul Islam: conceptualization, supervision, validation, and writing—review and editing.

## Funding

Open access publishing facilitated by La Trobe University, as part of the Wiley ‐ La Trobe University agreement via the Council of Australasian University Librarians.

## Conflicts of Interest

The authors declare no conflicts of interest.

## Supporting information


**Supporting Information** Additional supporting information can be found online in the Supporting Information section. Quality assessment of 19 papers using the Mixed Methods Appraisal Tool (MMAT), Version 2018.

## Data Availability

The authors confirm that the data supporting the findings of this study are available within the article and its supporting information.
