# Supplementary material for: Understanding Barriers to Disclosure of and Treatment for Substance Use During Pregnancy: A Narrative Synthesis of Literature
Source: J Pregnancy. 2026 Jul 15;2026:6969976. doi: 10.1155/jp/6969976 (PMC13370109; doi:10.1155/jp/6969976)
Supplement: Supplementary file 1 — Supporting Information Additional supporting information can be found online in the Supporting Information section. Quality assessment of 19 papers using the Mixed Methods Appraisal Tool (MMAT), Version 2018. [file JP-2026-6969976-s001.docx]

**Supplementary material:**

**Quality assessment of 19 papers using the Mixed Methods Appraisal Tool (MMAT), version 2018**

| **Pregnant women and substance use: fear, stigma, and barriers to care** | | | | | |
| --- | --- | --- | --- | --- | --- |
| **Category of study designs** | **Methodological quality criteria** | **Responses** | | | |
|  |  | Yes | No | Can’t tell | Comments |
| Screening questions (for all types) | S1. Are there clear research questions? | P |  |  |  |
|  | S2. Do the collected data allow to address the research questions? | P |  |  |  |
|  | *Further appraisal may not be feasible or appropriate when the answer is ‘No’ or ‘Can’t tell’ to one or both screening questions.* | | | | |
| 1. Qualitative | 1.1. Is the qualitative approach appropriate to answer the research question? | P |  |  |  |
|  | 1.2. Are the qualitative data collection methods adequate to address the research question? | P |  |  |  |
|  | 1.3. Are the findings adequately derived from the data? | P |  |  |  |
|  | 1.4. Is the interpretation of results sufficiently substantiated by data? | P |  |  |  |
|  | 1.5. Is there coherence between qualitative data sources, collection, analysis and interpretation? | P |  |  |  |

| **Factors that influence Women’s disclosures of substance use during pregnancy: A qualitative study of Ten Midwifes and Ten pregnant women** | | | | | |
| --- | --- | --- | --- | --- | --- |
| **Category of study designs** | **Methodological quality criteria** | **Responses** | | | |
|  |  | Yes | No | Can’t tell | Comments |
| Screening questions (for all types) | S1. Are there clear research questions? | P |  |  |  |
|  | S2. Do the collected data allow to address the research questions? | P |  |  |  |
|  | *Further appraisal may not be feasible or appropriate when the answer is ‘No’ or ‘Can’t tell’ to one or both screening questions.* | | | | |
| 1. Qualitative | 1.1. Is the qualitative approach appropriate to answer the research question? | P |  |  |  |
|  | 1.2. Are the qualitative data collection methods adequate to address the research question? | P |  |  |  |
|  | 1.3. Are the findings adequately derived from the data? | P |  |  |  |
|  | 1.4. Is the interpretation of results sufficiently substantiated by data? | P |  |  |  |
|  | 1.5. Is there coherence between qualitative data sources, collection, analysis and interpretation? | P |  |  |  |

| **Psychometric properties of the prenatal opioid use perceived stigma scale and its use in prenatal care** | | | | | |
| --- | --- | --- | --- | --- | --- |
| **Category of study designs** | **Methodological quality criteria** | **Responses** | | | |
|  |  | Yes | No | Can’t tell | Comments |
| Screening questions (for all types) | S1. Are there clear research questions? | P |  |  |  |
|  | S2. Do the collected data allow to address the research questions? | P |  |  |  |
|  | *Further appraisal may not be feasible or appropriate when the answer is ‘No’ or ‘Can’t tell’ to one or both screening questions.* | | | | |
| 1. Quantitative | 1.1. Is the sampling strategy relevant to address the research question? | P |  |  |  |
|  | 1.2. Is the sample representative of the target population? | P |  |  |  |
|  | 1.3. Are the measurements appropriate? |  |  | P | The text remains silent on whether the questionnaires were pre-tested, but the mention of validation implies a level of careful development |
|  | 1.4. Is the risk of nonresponse bias low? |  |  | P | The study does not provide specific information on the nonresponse rate. |
|  | 1.5. Is the statistical analysis appropriate to answer the research question? | P |  |  |  |

| **Social stigma and perinatal substance use services: Recognizing the power of the good mother ideal** | | | | | |
| --- | --- | --- | --- | --- | --- |
| **Category of study designs** | **Methodological quality criteria** | **Responses** | | | |
|  |  | Yes | No | Can’t tell | Comments |
| Screening questions (for all types) | S1. Are there clear research questions? | P |  |  |  |
|  | S2. Do the collected data allow to address the research questions? | P |  |  |  |
|  | *Further appraisal may not be feasible or appropriate when the answer is ‘No’ or ‘Can’t tell’ to one or both screening questions.* | | | | |
| 1. Qualitative | 1.1. Is the qualitative approach appropriate to answer the research question? | P |  |  |  |
|  | 1.2. Are the qualitative data collection methods adequate to address the research question? | P |  |  |  |
|  | 1.3. Are the findings adequately derived from the data? | P |  |  |  |
|  | 1.4. Is the interpretation of results sufficiently substantiated by data? | P |  |  |  |
|  | 1.5. Is there coherence between qualitative data sources, collection, analysis and interpretation? | P |  |  |  |

| **Complex calculations: how drug use during pregnancy becomes a barrier to prenatal care** | | | | | |
| --- | --- | --- | --- | --- | --- |
| **Category of study designs** | **Methodological quality criteria** | **Responses** | | | |
|  |  | Yes | No | Can’t tell | Comments |
| Screening questions (for all types) | S1. Are there clear research questions? | P |  |  |  |
|  | S2. Do the collected data allow to address the research questions? | P |  |  |  |
|  | *Further appraisal may not be feasible or appropriate when the answer is ‘No’ or ‘Can’t tell’ to one or both screening questions.* | | | | |
| 1. Qualitative | 1.1. Is the qualitative approach appropriate to answer the research question? | P |  |  |  |
|  | 1.2. Are the qualitative data collection methods adequate to address the research question? | P |  |  |  |
|  | 1.3. Are the findings adequately derived from the data? | P |  |  |  |
|  | 1.4. Is the interpretation of results sufficiently substantiated by data? | P |  |  |  |
|  | 1.5. Is there coherence between qualitative data sources, collection, analysis and interpretation? | P |  |  |  |

| **In their own words: A qualitative study of factors promoting resilience and recovery among postpartum women with opioid use disorders** | | | | | |
| --- | --- | --- | --- | --- | --- |
| **Category of study designs** | **Methodological quality criteria** | **Responses** | | | |
|  |  | Yes | No | Can’t tell | Comments |
| Screening questions (for all types) | S1. Are there clear research questions? | P |  |  |  |
|  | S2. Do the collected data allow to address the research questions? | P |  |  |  |
|  | *Further appraisal may not be feasible or appropriate when the answer is ‘No’ or ‘Can’t tell’ to one or both screening questions.* | | | | |
| 1. Qualitative | 1.1. Is the qualitative approach appropriate to answer the research question? | P |  |  |  |
|  | 1.2. Are the qualitative data collection methods adequate to address the research question? | P |  |  |  |
|  | 1.3. Are the findings adequately derived from the data? | P |  |  | The study did not thoroughly investigate potential negative cases or contradictory experiences, despite the findings being largely consistent with the data. The credibility of the results could have been improved by including an analysis of both successful and unsuccessful recovery cases, rather than focusing solely on resilience and positive outcomes |
|  | 1.4. Is the interpretation of results sufficiently substantiated by data? | P |  |  |  |
|  | 1.5. Is there coherence between qualitative data sources, collection, analysis and interpretation? | P |  |  |  |

| **The experience of pregnancy, childbirth and motherhood of drug-using women** | | | | | |
| --- | --- | --- | --- | --- | --- |
| **Category of study designs** | **Methodological quality criteria** | **Responses** | | | |
|  |  | Yes | No | Can’t tell | Comments |
| Screening questions (for all types) | S1. Are there clear research questions? | P |  |  |  |
|  | S2. Do the collected data allow to address the research questions? | P |  |  |  |
|  | *Further appraisal may not be feasible or appropriate when the answer is ‘No’ or ‘Can’t tell’ to one or both screening questions.* | | | | |
| 1. Qualitative | 1.1. Is the qualitative approach appropriate to answer the research question? | P |  |  |  |
|  | 1.2. Are the qualitative data collection methods adequate to address the research question? | P |  |  |  |
|  | 1.3. Are the findings adequately derived from the data? | P |  |  |  |
|  | 1.4. Is the interpretation of results sufficiently substantiated by data? | P |  |  |  |
|  | 1.5. Is there coherence between qualitative data sources, collection, analysis and interpretation? |  |  | P | While the study demonstrates a general coherence between data collection, analysis and interpretation, there are areas where deeper triangulation, critical engagement with participant narratives, and broader integration of structural factors could strengthen the alignment. |

| **Barriers to women's disclosure of and treatment for substance use during pregnancy: A qualitative study** | | | | | |
| --- | --- | --- | --- | --- | --- |
| **Category of study designs** | **Methodological quality criteria** | **Responses** | | | |
|  |  | Yes | No | Can’t tell | Comments |
| Screening questions (for all types) | S1. Are there clear research questions? | P |  |  |  |
|  | S2. Do the collected data allow to address the research questions? | P |  |  |  |
|  | *Further appraisal may not be feasible or appropriate when the answer is ‘No’ or ‘Can’t tell’ to one or both screening questions.* | | | | |
| 1. Qualitative | 1.1. Is the qualitative approach appropriate to answer the research question? | P |  |  |  |
|  | 1.2. Are the qualitative data collection methods adequate to address the research question? | P |  |  |  |
|  | 1.3. Are the findings adequately derived from the data? | P |  |  |  |
|  | 1.4. Is the interpretation of results sufficiently substantiated by data? | P |  |  |  |
|  | 1.5. Is there coherence between qualitative data sources, collection, analysis and interpretation? | P |  |  |  |

| **The labor and birth experience of women with opioid use disorder: A qualitative study** | | | | | |
| --- | --- | --- | --- | --- | --- |
| **Category of study designs** | **Methodological quality criteria** | **Responses** | | | |
|  |  | Yes | No | Can’t tell | Comments |
| Screening questions (for all types) | S1. Are there clear research questions? | P |  |  |  |
|  | S2. Do the collected data allow to address the research questions? | P |  |  |  |
|  | *Further appraisal may not be feasible or appropriate when the answer is ‘No’ or ‘Can’t tell’ to one or both screening questions.* | | | | |
| 1. Qualitative | 1.1. Is the qualitative approach appropriate to answer the research question? | P |  |  |  |
|  | 1.2. Are the qualitative data collection methods adequate to address the research question? | P |  |  |  |
|  | 1.3. Are the findings adequately derived from the data? | P |  |  |  |
|  | 1.4. Is the interpretation of results sufficiently substantiated by data? | P |  |  |  |
|  | 1.5. Is there coherence between qualitative data sources, collection, analysis and interpretation? | P |  |  |  |

| **Health Care Providers' Perceived Barriers to Screening for Substance Use During Pregnancy** | | | | | |
| --- | --- | --- | --- | --- | --- |
| **Category of study designs** | **Methodological quality criteria** | **Responses** | | | |
|  |  | Yes | No | Can’t tell | Comments |
| Screening questions (for all types) | S1. Are there clear research questions? | P |  |  |  |
|  | S2. Do the collected data allow to address the research questions? | P |  |  |  |
|  | *Further appraisal may not be feasible or appropriate when the answer is ‘No’ or ‘Can’t tell’ to one or both screening questions.* | | | | |
| 1. Qualitative | 1.1. Is the qualitative approach appropriate to answer the research question? | P |  |  |  |
|  | 1.2. Are the qualitative data collection methods adequate to address the research question? | P |  |  |  |
|  | 1.3. Are the findings adequately derived from the data? | P |  |  | The findings could be more robust if they included a more systematic thematic analysis of the qualitative data. |
|  | 1.4. Is the interpretation of results sufficiently substantiated by data? | P |  |  |  |
|  | 1.5. Is there coherence between qualitative data sources, collection, analysis and interpretation? |  | P |  |  |

| **Secrecy Versus Disclosure: Women with Substance Use Disorders Share Experiences in Help Seeking During Pregnancy** | | | | | |
| --- | --- | --- | --- | --- | --- |
| **Category of study designs** | **Methodological quality criteria** | **Responses** | | | |
|  |  | Yes | No | Can’t tell | Comments |
| Screening questions (for all types) | S1. Are there clear research questions? | P |  |  |  |
|  | S2. Do the collected data allow to address the research questions? | P |  |  |  |
|  | *Further appraisal may not be feasible or appropriate when the answer is ‘No’ or ‘Can’t tell’ to one or both screening questions.* | | | | |
| 1. Qualitative | 1.1. Is the qualitative approach appropriate to answer the research question? | P |  |  |  |
|  | 1.2. Are the qualitative data collection methods adequate to address the research question? | P |  |  |  |
|  | 1.3. Are the findings adequately derived from the data? | P |  |  |  |
|  | 1.4. Is the interpretation of results sufficiently substantiated by data? | P |  |  |  |
|  | 1.5. Is there coherence between qualitative data sources, collection, analysis and interpretation? | P |  |  |  |

| **Conceptualizing stigma in contexts of pregnancy and opioid misuse: A qualitative study with women and healthcare providers in Ohio** | | | | | |
| --- | --- | --- | --- | --- | --- |
| **Category of study designs** | **Methodological quality criteria** | **Responses** | | | |
|  |  | Yes | No | Can’t tell | Comments |
| Screening questions (for all types) | S1. Are there clear research questions? | P |  |  |  |
|  | S2. Do the collected data allow to address the research questions? | P |  |  |  |
|  | *Further appraisal may not be feasible or appropriate when the answer is ‘No’ or ‘Can’t tell’ to one or both screening questions.* | | | | |
| 1. Qualitative | 1.1. Is the qualitative approach appropriate to answer the research question? | P |  |  |  |
|  | 1.2. Are the qualitative data collection methods adequate to address the research question? | P |  |  |  |
|  | 1.3. Are the findings adequately derived from the data? | P |  |  |  |
|  | 1.4. Is the interpretation of results sufficiently substantiated by data? | P |  |  |  |
|  | 1.5. Is there coherence between qualitative data sources, collection, analysis and interpretation? | P |  |  |  |

| “**The elephant in the room," a qualitative study of perinatal fears in opioid use disorder treatment in Southern Appalachia** | | | | | |
| --- | --- | --- | --- | --- | --- |
| **Category of study designs** | **Methodological quality criteria** | **Responses** | | | |
|  |  | Yes | No | Can’t tell | Comments |
| Screening questions (for all types) | S1. Are there clear research questions? | P |  |  |  |
|  | S2. Do the collected data allow to address the research questions? | P |  |  |  |
|  | *Further appraisal may not be feasible or appropriate when the answer is ‘No’ or ‘Can’t tell’ to one or both screening questions.* | | | | |
| 1. Qualitative | 1.1. Is the qualitative approach appropriate to answer the research question? | P |  |  |  |
|  | 1.2. Are the qualitative data collection methods adequate to address the research question? | P |  |  |  |
|  | 1.3. Are the findings adequately derived from the data? | P |  |  |  |
|  | 1.4. Is the interpretation of results sufficiently substantiated by data? | P |  |  |  |
|  | 1.5. Is there coherence between qualitative data sources, collection, analysis and interpretation? | P |  |  |  |

| **Treatment for substance use disorders in pregnant women: Motivators and barriers** | | | | | |
| --- | --- | --- | --- | --- | --- |
| **Category of study designs** | **Methodological quality criteria** | **Responses** | | | |
|  |  | Yes | No | Can’t tell | Comments |
| Screening questions (for all types) | S1. Are there clear research questions? | P |  |  |  |
|  | S2. Do the collected data allow to address the research questions? | P |  |  |  |
|  | *Further appraisal may not be feasible or appropriate when the answer is ‘No’ or ‘Can’t tell’ to one or both screening questions.* | | | | |
| 1. Qualitative | 1.1. Is the qualitative approach appropriate to answer the research question? | P |  |  |  |
|  | 1.2. Are the qualitative data collection methods adequate to address the research question? | P |  |  |  |
|  | 1.3. Are the findings adequately derived from the data? | P |  |  |  |
|  | 1.4. Is the interpretation of results sufficiently substantiated by data? | P |  |  |  |
|  | 1.5. Is there coherence between qualitative data sources, collection, analysis and interpretation? | P |  |  |  |

| **Personas of pregnant and parenting women with substance use and their barriers and pathways to system engagement** | | | | | |
| --- | --- | --- | --- | --- | --- |
| **Category of study designs** | **Methodological quality criteria** | **Responses** | | | |
|  |  | Yes | No | Can’t tell | Comments |
| Screening questions (for all types) | S1. Are there clear research questions? | P |  |  |  |
|  | S2. Do the collected data allow to address the research questions? | P |  |  |  |
|  | *Further appraisal may not be feasible or appropriate when the answer is ‘No’ or ‘Can’t tell’ to one or both screening questions.* | | | | |
| 1. Qualitative | 1.1. Is the qualitative approach appropriate to answer the research question? | P |  |  |  |
|  | 1.2. Are the qualitative data collection methods adequate to address the research question? | P |  |  |  |
|  | 1.3. Are the findings adequately derived from the data? | P |  |  |  |
|  | 1.4. Is the interpretation of results sufficiently substantiated by data? | P |  |  |  |
|  | 1.5. Is there coherence between qualitative data sources, collection, analysis and interpretation? | P |  |  |  |

| **Pregnant people’s experiences discussing their cannabis use with prenatal care providers in a state with legalized cannabis** | | | | | |
| --- | --- | --- | --- | --- | --- |
| **Category of study designs** | **Methodological quality criteria** | **Responses** | | | |
|  |  | Yes | No | Can’t tell | Comments |
| Screening questions (for all types) | S1. Are there clear research questions? | P |  |  |  |
|  | S2. Do the collected data allow to address the research questions? | P |  |  |  |
|  | *Further appraisal may not be feasible or appropriate when the answer is ‘No’ or ‘Can’t tell’ to one or both screening questions.* | | | | |
| 1. Qualitative | 1.1. Is the qualitative approach appropriate to answer the research question? | P |  |  |  |
|  | 1.2. Are the qualitative data collection methods adequate to address the research question? | P |  |  |  |
|  | 1.3. Are the findings adequately derived from the data? | P |  |  |  |
|  | 1.4. Is the interpretation of results sufficiently substantiated by data? | P |  |  |  |
|  | 1.5. Is there coherence between qualitative data sources, collection, analysis and interpretation? | P |  |  |  |

| **Pregnancy- and parenting-related barriers to receiving medication for opioid use disorder: A multi-paneled qualitative study of women in treatment, women who terminated treatment, and the professionals who serve them** | | | | | |
| --- | --- | --- | --- | --- | --- |
| **Category of study designs** | **Methodological quality criteria** | **Responses** | | | |
|  |  | Yes | No | Can’t tell | Comments |
| Screening questions (for all types) | S1. Are there clear research questions? | P |  |  |  |
|  | S2. Do the collected data allow to address the research questions? | P |  |  |  |
|  | *Further appraisal may not be feasible or appropriate when the answer is ‘No’ or ‘Can’t tell’ to one or both screening questions.* | | | | |
| 1. Qualitative | 1.1. Is the qualitative approach appropriate to answer the research question? | P |  |  |  |
|  | 1.2. Are the qualitative data collection methods adequate to address the research question? | P |  |  |  |
|  | 1.3. Are the findings adequately derived from the data? | P |  |  |  |
|  | 1.4. Is the interpretation of results sufficiently substantiated by data? | P |  |  |  |
|  | 1.5. Is there coherence between qualitative data sources, collection, analysis and interpretation? | P |  |  |  |

| **Barriers to care for pregnant women seeking substance use disorder treatment** | | | | | |
| --- | --- | --- | --- | --- | --- |
| **Category of study designs** | **Methodological quality criteria** | **Responses** | | | |
|  |  | Yes | No | Can’t tell | Comments |
| Screening questions (for all types) | S1. Are there clear research questions? | P |  |  |  |
|  | S2. Do the collected data allow to address the research questions? | P |  |  |  |
|  | *Further appraisal may not be feasible or appropriate when the answer is ‘No’ or ‘Can’t tell’ to one or both screening questions.* | | | | |
| 1. Qualitative | 1.1. Is the qualitative approach appropriate to answer the research question? | P |  |  |  |
|  | 1.2. Are the qualitative data collection methods adequate to address the research question? | P |  |  |  |
|  | 1.3. Are the findings adequately derived from the data? | P |  |  |  |
|  | 1.4. Is the interpretation of results sufficiently substantiated by data? | P |  |  |  |
|  | 1.5. Is there coherence between qualitative data sources, collection, analysis and interpretation? | P |  |  |  |

| **Extrinsic barriers to Substance abuse treatment among Pregnant drug dependent women** | | | | | |
| --- | --- | --- | --- | --- | --- |
| **Category of study designs** | **Methodological quality criteria** | **Responses** | | | |
|  |  | Yes | No | Can’t tell | Comments |
| Screening questions (for all types) | S1. Are there clear research questions? | P |  |  |  |
|  | S2. Do the collected data allow to address the research questions? | P |  |  |  |
|  | *Further appraisal may not be feasible or appropriate when the answer is ‘No’ or ‘Can’t tell’ to one or both screening questions.* | | | | |
| 1. Qualitative | 1.1. Is the qualitative approach appropriate to answer the research question? | P |  |  |  |
|  | 1.2. Are the qualitative data collection methods adequate to address the research question? | P |  |  |  |
|  | 1.3. Are the findings adequately derived from the data? | P |  |  |  |
|  | 1.4. Is the interpretation of results sufficiently substantiated by data? | P |  |  |  |
|  | 1.5. Is there coherence between qualitative data sources, collection, analysis and interpretation? | P |  |  |  |
